# A secondary qualitative analysis of stakeholder views about participant recruitment, retention, and adherence in decentralised clinical trials (DCTs)

**DOI:** 10.1186/s13063-022-06521-4

**Published:** 2022-07-30

**Authors:** Joanne Coyle, Amy Rogers, Rachel Copland, Giorgia De Paoli, Thomas M. MacDonald, Isla S. Mackenzie, on behalf of the Trials@Home Consortium

**Affiliations:** MEMO Research, Division of Molecular and Clinical Medicine, Ninewells Hospital and Medical School, University of Dundee, Dundee, DD1 9SY UK

**Keywords:** Decentralised clinical trials, DCT, Virtual trials, Recruitment, Retention, Adherence, Qualitative interviews

## Abstract

**Background:**

Decentralised clinical trials (DCTs) are clinical trials where all or most trial activities occur in or near participants’ homes instead of hospitals or research sites. While more convenient for participants, DCTs may offer limited opportunities to build trust with investigators and trial teams. This qualitative analysis explored DCT stakeholder views to inform strategies for maximising participant recruitment, retention, and adherence.

**Methods:**

A secondary analysis of original interview transcripts focused on participant engagement: recruitment, retention, and adherence. Semi-structured interviews were conducted with a purposive sample of stakeholders, including trial managers and administrators, investigators, nurses, vendors, and patient representatives. Interview data were coded using a thematic approach to generate descriptive themes.

**Results:**

Forty-eight stakeholders were interviewed. Three components of participant engagement in DCTs were identified: identifying and attracting potential participants, retaining participants and encouraging adherence, and involvement of patients and the public. Interviewees believed that a potential participant’s beliefs about research value and their trust in the research team strongly influenced the likelihood of taking part in a DCT. Early involvement of patients was identified as one way to gauge participant priorities. However, perceived burden was seen as a barrier to recruitment. Factors influencing retention and adherence were related to the same underlying motivators that drove recruitment: personal values, circumstances, and burden. Being part of a DCT should not conflict with the original motivations to participate.

**Conclusion:**

Recruitment, retention, and adherence in DCTs are driven by factors that have previously been found to affect conventional clinical trials. Increasing patient and public involvement can address many of these factors. In contrast to conventional trials, DCTs are perceived as requiring greater emphasis on communication, and contact, to engender trust between participants and researchers despite a relative lack of in-person interaction.

## Background

Successful clinical trials rely on their ability to recruit participants. However, trials must also retain participants and encourage adherence to required trial activities. This article explores the understanding of recruitment, retention, and engagement in decentralised clinical trials (DCTs) through a secondary analysis of interview transcript data collected to investigate stakeholder experiences of DCTs [1].

DCTs are clinical trials where all or most trial activities occur in or near participants’ homes instead of hospitals or research sites; they have been purported to improve patient-centredness [2]. However, DCTs, while potentially more convenient, may offer limited opportunities for building relationships through social interactions between patients and healthcare professionals and between participants and researchers, typical of conventional site-based trials [2, 3]. It is unclear whether this relative lack of social contact may impact upon people’s willingness to participate in DCTs; for example, by interacting differently with motivators that drive trial participation. In a 2020 systematic review, Sheridan et al. identified the dominant motivators for research participation as “potential for personal benefit, altruism, and trust” [4]. If trust in clinical trials relies upon interpersonal interactions, will DCTs struggle to recruit and retain?

During our earlier analysis, we noted that the term “patient engagement” was frequently used by people working in DCTs to refer to activities aimed at improving recruitment, retention, and adherence [1]. These activities could range from advertising opportunities to participate to specific operational features to stimulate recruitment and maintain active and sustained participation [5]. A vital element of this usage is that it concerns participants in specific trials rather than a larger group of patients who may or may not be trial participants. In this article, we use “participant engagement” to refer to activities aiming to improve recruitment, retention, or adherence by interacting with potential or actual trial participants. A specific challenge that has been identified for DCTs is to maximise participant engagement despite the apparent limitations on communication and relationship building presented by the relative lack of in-person researcher-participant interaction [6].

There are significant implications for DCTs that fail to recruit and retain participants. Inadequate recruitment may prolong a trial or prevent it from proceeding. Even where remote studies have achieved impressive recruitment, this does not automatically translate into sustained adherence to trial activities and retention of participants, as evident in the Apple Watch Study [7]. Once recruited, there remain challenges in supporting participants to adhere to protocol-required activities; poor adherence can cause missing data that may bias study results. In addition, early study withdrawals can cause problems with missing outcome data or costly requirements to lengthen follow-up.

Robust evidence to support effective recruitment, retention, and adherence in clinical trials is limited [8–10]. However, involving patients in activities such as identifying research priorities, reviewing study protocols, and developing participant information sheets and other materials can be beneficial [3]. Indeed, a systematic review reported that this kind of activity, referred to as “patient engagement”, improves study enrolment [11]. Additionally, patient advisory panels and focus groups have been found to have the lowest cost and highest impact on clinical research and development relative to other patient-centric initiatives [12].

The term “patient engagement” is widely used in healthcare and healthcare research and has been referred to as key to the success of clinical trials [13]. Despite this wide adoption, accepted healthcare engagement definitions differ from the observed clinical trial usage we noted above. A review by the Patient Engagement in Research Working Group of the ISPOR (International Society for Pharmacoeconomics and Outcomes Research) Patient-Centered Special Interest Group aimed to generate a robust definition of patient engagement that could be used in health research as follows:

The active, meaningful, and collaborative interaction between patients and researchers across all stages of the research process, where research decision-making is guided by patients’ contributions as partners, recognising their specific experiences, values and expertise [14].

In addition to exploring aspects of recruitment, retention, and adherence as reported by the interviewed stakeholders, this article also investigates the interaction between “patient engagement”, as defined by ISPOR, and participant engagement (i.e. recruitment, retention, and adherence) in DCTs.

The Trials@Home project (https://trialsathome.com) is a multi-stakeholder project supported by the EU/EFPIA (European Federation of Pharmaceutical Industries and Associations) Innovative Medicines Initiative. The research aims to learn from representatives of academic institutions, pharmaceutical companies, small-medium enterprises, and patient representatives about their experiences developing and implementing DCT methods. The secondary analysis described in this paper is an extension of a previous primary analysis with a conceptual focus on participant engagement in DCTs.

## Methods

During our earlier analysis, we observed several interviewees referring to “engagement” as an essential consideration in DCTs but with varying apparent usage of the term. Using transcripts of interviews with DCT-involved stakeholders, we explored how interviewees spoke about participant engagement when talking about their DCT experiences and what they understood “engagement” to mean in the context of DCTs.

The methods used to collect the data analysed in this paper have been previously described [1]. Here is a summary.

### Design and study setting

We conducted one-to-one semi-structured interviews with stakeholders involved in 20 DCT case studies. This report follows the Consolidated criteria for reporting qualitative studies (COREQ) 32-item checklist guidance [15].

### Participants and sample

#### Case study identification and selection

DCT case studies were purposefully selected to represent various methods and therapeutic areas (Table 1).

**Table 1:** Characteristics of included case studies

Case study characteristics^**a**^	***n***
Case studies	20
DCT type	
Hybrid	14
Remote	6
Therapeutic area	
Cardiovascular	6
Diabetes	4
Rheumatology	3
Neurology	3
Women’s health	2
Others	2
Location of the lead investigator	
North America	11
UK	7
Mainland Europe	2
Location of participants	
Single country	15
International	5
Status (at time of interview)	
Ongoing	11
Completed	7
In set-up phase	2

^a^For more detailed information on the included case studies, please see [1]

#### Selection of participants

Case study proposers were asked to generate a list of potential interviewees for invitation. Participants were purposively sampled to capture a diversity of opinions and experiences.

#### Recruitment

Forty-eight participants were recruited between December 2019 and June 2020. Reasons for declining participation included trials still in early set-up phases and diversion of staff to COVID-19 work. Participants did not receive any remuneration and had various roles (see the “Results” section).

### Instrument development

Using an empirical phenomenology approach, we developed a semi-structured interview schema with open questions to encourage interviewees to describe and ascribe meaning to their experiences with DCTs, focusing on challenges, solutions, and learnings [16].

### Interview procedure

All interviews were carried out by an experienced qualitative researcher (JC) based in the UK between 15 January 2020 and 26 June 2020. Twelve interviews were conducted in person, 35 by videoconferencing, and one by telephone. Interviews were 1 h long, on average, and were audio-recorded and transcribed.

### Data analysis

We used a thematic analysis approach [17]; this involved familiarisation, generating initial codes, developing preliminary descriptive themes, reviewing and modifying themes, and final refining.

#### Primary analysis

Two co-authors (a qualitative researcher and clinician-researcher) independently read the transcripts and generated initial codes and categories. Similarities, differences, and clustering were noted, reaching an agreement on initial descriptive themes. These were shared with the remaining authors and the wider research team for further refinement until consensus was achieved.

#### Secondary analysis

The secondary analysis presented here extends the primary analysis with a conceptual focus on engagement. It was conducted by the qualitative researcher, who identified existing relevant codes and reviewed the transcripts, re-coding where necessary. New themes were developed and discussed with the clinician-researcher before discussion with the broader authorship. We explored how interviewees described engaging participants with specific attention to recruitment, retention, and encouraging adherence to trial activities. We also sought to identify any DCT-specific challenges to achieving engagement.

### Role of the funding source

This research was funded by an EU/EFPIA Innovative Medicines Initiative (H2020-JTI-IMI2, grant no. 831458) as part of the Trials@Home Centre of Excellence for Remote and Decentralized Clinical Trials (https://trialsathome.com). Trials@Home consortium partners helped identify case studies and participants and provided feedback on interim findings. The funder had no role in the design and conduct of the qualitative research or interpretation of the data. The manuscript was reviewed and approved for submission by the Trials@Home Partner Assembly and Co-ordination Team.

## Results

Forty-eight stakeholders from 20 case studies were interviewed, including 19 clinical/research trial staff, 17 management/administration trial staff, 6 technology/data trial staff, 4 vendors, and 2 patient representatives.

Three domains of participant engagement activity were identified in the secondary analysis: identifying and attracting potential participants (recruitment), retaining participants and encouraging their adherence to trial activities, and involving patients and the public. Interviewees who worked on DCTs frequently described performing patient and public involvement activities to inform and support recruitment, retention, and adherence. However, interviewees who had been participants in clinical trials referred to involvement activities as improving research more generally. Across the three activity domains, we constructed three overarching themes: perceived value of DCT participation, burden of DCT participation, and trust (between participants, researchers, and usual healthcare providers).

These themes are presented in Table 2, with illustrative quotes in Table 3, and are further elaborated below in relation to the identified participant engagement activity domains.

**Table 2:** Themes developed from interviewees’ descriptions of DCT participant engagement activities

Overarching themes	Sub-themes
Perceived value of DCT participation	Relatability of study aims
	Useful feedback
	Targeted recruitment
Burden of DCT participation	Familiarity with study activities and technology
	Simple instructions and interfaces
	Participatory choice
Trust	Communication
	Maintaining contact

**Table 3:** Selected quotations illustrating the themes developed from interviewees’ descriptions of DCT participant engagement activities

Theme	Illustrative quotations
Sub-theme	
**Perceived value of DCT participation**	
Relatability of study aims	“The recruitment rate was so high. So, we got an astounding response rate to a cold call letter…And it’s because of the subject, it's because it’s retinopathy, blindness is the number one fear of people with diabetes and these are people that have been told they’ve got changes to their eyes and there’s nothing we can do about it…And that must be terrifying.” Case study interviewee 0049 Trial Staff (clinical/research) “We also put quite a lot of work into our websites ahead of time and we’ve got a video on the front page from Fred McCauley who I knew used to be on BBC Scotland for years and I know he’s still doing the tour as a comedian.” Case study interviewee 0041 Trial Staff (clinical/research)
Useful feedback	“But the other thing also, the measure is satisfaction by the patients, we have a very comprehensive survey about that. We measured what features are important to them and how well did we do in that category? So, we discovered that, for example, that being able to see your own measures was really highly valued.” *Case study interviewee 0015 Trial Staff (technology/data)*
Targeted recruitment	“My role…was really to go and recruit the patients, first of all, from lots of different GP practices. So, we had to go out and do a search so the IT team had a search that we could go out and run on the computers, so it was actually on, I think, a portal….Then once we found the patients, then we had to send out letters… Then we had check the replies, then…The ones that had wanted to take part, we would get in contact with them, either by phone or email and then we would need to go and organise to go back to the practice and to screen these patients.” *Interviewee 0008 Trial Staff (clinical/research)* “…Recruitment was done all through social media and we contacted support groups, and we the head representatives go out, they have an annual, one of the support groups has an annual conference so they went out, they set up a booth...” *Case study interviewee 0021 Trial Staff (clinical/research)*
**Burden of DCT participation**	“We said, why don’t we try throwing the kitchen sink at it? So, let’s do remote tele-health monitoring with some virtual devices. We had the patients take their blood pressure remotely, take their weight, their pulse, the glucose metre and… we developed an app for the study, the patients would use on their phone and the app had many functions but basically the patient could log on every day, do their e-diary and that would be reviewed when they were doing their remote vital signs, their other protocol procedures…it’s a lot” *Case study interviewee 0044 Trial Staff (management/administration)* “We got their feedback on the devices… nobody really loved the biosensor, that they had to wear it stuck on your chest, it had flashing lights on it, you could see the lights through your shirt.” *Case study interviewee 0053 Vendor*
Familiarity with study activities and technology	“So, they were not always necessarily familiar with the technology, right? And so that could have been, could be a reason for challenging patient recruitment.” *Case study interviewee 0029 Trial Staff (management/administration)*
Simple instructions and interfaces	“Yeah, so that was all in the patient portal. So, in the patient portal there was a graphic that showed them where they are, what to do next…Then there was a messaging function. So, through the messaging function there was a secure chat in the portal that they could use to reach out to the study team. They also had a phone mechanism to call if they have a question.” *Case study interviewee 0015 Trial Staff (technology/data)* “In the beginning we also had some issues and, for example of a bad practice, so when you go into the online environment people can easily misunderstand things and you have to be very careful with your wording… So, one of the questions we had in the pre-screener which made a lot of sense to us but not to our users.” *Case study interviewee 0015 Trial Staff (technology/data)*
Participatory choice	“So, for patients that were comfortable using the internet they were able to complete the visits on their own. We didn’t want to restrict in a socioeconomic way…So there is a way for patients once they are enrolled for the central call centre at [...] to call and help facilitate completion of follow up visits. Just so that the patient either did not have consistent access to the internet, or was not comfortable using the internet, that they still had a method for participating in the study.” *Case study interviewee 0016 Trial Staff (management/administration)*
**Trust**	“What we found is successful…is even in a virtual study patients want to know that their clinicians…support them during the clinical study and support them doing something different… So even in a virtual study where you are able to identify patients probably you still need to engage clinicians and have that support and buy-in and be able to support that relationship between eligible patients and their providers.” *Case study interviewee 0016 Trial Staff (management/administration)*
Communication	“Our patient partners were also really critical in the design of the recruitment materials. So, again you think a lot of recruitment materials at least in the US, there's a lot of language… it's got a lot of text…they felt that if we were going to be approaching patients remotely, either through email or through regular mail, phone calls that we really had to let a patient know very quickly this is a clinical study, this is the goal of the clinical study and who we are looking to enrol...” *Case study interviewee 0016 Trial Staff (management/administration)*
Maintaining contact	“We made sure that we would help retention by keeping contact with the patient. They received letters from us every second month, they received Christmas greeting cards, newsletters, stuff like that. And we contacted them whenever there was suspicion of an end point.” Case study interviewee 0017 Trial Staff (clinical/research) “Then for retention, we talked to our patient partners about how do we make sure that patients want to come back and complete follow-up visits? How do we keep them engaged when they are not going into the clinic and seeing people and building that personal relationship? And so, we developed a participant newsletter, and our patient partners helped to develop the different sections of the newsletters.” Case study interviewee 0016 Trial Staff (management/administration)

### Identifying and attracting potential participants

Interviewees who worked as clinical or administrative DCT staff frequently talked about recruitment activities when asked how they had engaged with their participants.

#### Perceived value of DCT participation

Several interviewees believed their DCT had achieved a high recruitment rate because patients believed their research was of particular value to them. Perceived value was thought to be related to a participant’s perception of threat (of their condition) and their beliefs about the efficacy of existing treatment choices. Many interviewees stressed that they needed to consider what motivated individuals to participate. For example, one interviewee explained how their trial’s focus on an eye complication of diabetes ensured a higher level of recruitment than expected:

Now, the approach that we used is similar to what we do for our cardiovascular trials….We use NHS data to do big mailouts… So, all I can conclude is that population concern about their eyes is substantially higher than they have about their [other] clinical conditions.

Case study interviewee 0041 Trial Staff (clinical/research)

Interviewees also suggested that participants could be motivated to take part in a DCT if they believed there were few treatment options currently available to them:

Up until recently, there were very, very few medications approved for treatment of cluster headaches. So patients were quite motivated to learn of new things and help us out in each study.

Case Study Interviewee 0021 Trial Staff (clinical/research)

##### Relatability of study aims 

Interviewees highlighted the value of trial materials that focused on people with whom potential participants could identify, e.g. one trial featured a video of a famous comedian on their homepage; another included real patient-partner stories in their recruitment literature.

##### Targeted recruitment

Some interviewees reported that initial attempts to recruit through social media were unsuccessful; this was especially the case for trials that aimed to recruit participants with specific characteristics. For example, one interviewee described a time-consuming disease-based social media strategy that did not achieve the desired ethnically diverse participation. These interviewees recommended approaching patient advocacy groups, physicians’ societies, and organisations holding healthcare data to target potentially eligible individuals.

#### Burden of DCT participation

While a patient may be willing to participate in a DCT they consider to be of high value, interviewees felt this could be outweighed if it appeared that the DCT would place an undue burden on participants.

##### Participatory choice

Offering different ways to participate in DCTs was believed to encourage participation by allowing participants to choose which suits them best:

…you do need to consider that not all people are going to be comfortable using the internet or will still want some type of interaction. So having the ability to have facilitated completion of visits through phone calls is a great way to…provide opportunities for a broader group of people to participate.

Case study interviewee 0016 Trial Staff (management/administration)

#### Trust

Several interviewees identified a need to build strong, trusting relationships with potential participants and their usual healthcare providers. For example, a trial involving pregnant women required research nurses to take blood samples from the women and their infants at birth in hospital and, later, at home. To facilitate trust, the nurse met with the participant and their healthcare provider on multiple occasions:

This all had to be prepared very well in advance by visiting the hospital where the baby was going to be born…visiting the site staff. Getting to know them because they would be actually handing over some of their responsibilities to a nurse.

Case study interviewee 0031 Trial Staff (management/administration)

Emphasis was also placed on building relationships with sites and staff who would be responsible for recruiting participants, as the following interviewee explains:

…we actually went out and did a site visit to almost every single site…and we met with the clinical teams,…operational teams… we needed to engage the sites, and we needed to show them that we were as committed as them, so we went on the roadshow for the better part of a year.

Case study interviewee 0016 Trial Staff (management/administration)

### Retaining participants and encouraging their adherence to trial activities

Interviewees across all stakeholder groups appeared to understand the term “engagement” to relate to encouraging active study participation and adherence to required study activities.

#### Perceived value of DCT participation

In addition to the value of a DCT in answering an important clinical question, interviewees described other benefits for participants that might encourage them to remain in a trial.

##### Feeding back information

DCT participants highly value tools or interfaces that give immediate feedback. For example, a fully remote diabetes trial found that participants appreciated a continuous glucose monitor. In addition, several interviewees said their participants welcomed regular information on the trial’s progress. However, one interviewee, previously a participant in a conventional trial, explained the impact of not receiving such information:

One never got information back from it. One would go to hospital…every time you had your blood taken and weight and all kinds of stuff….But you never got any kind of results, any kind of inkling. And you never got anything at all at the end of it. …I thought [it] was pretty bad.

Case study interviewee 0058 Patient Representative

#### Burden of DCT participation

Several interviewees felt their trials overburdened patients with technology. For example, one interviewee believed their participants gradually disengaged and became less adherent due to the perceived burden of trial activities, reporting that they were required to perform several actions. Although the time necessary to perform these activities was not onerous, the perception of burden was high. This phenomenon was observed in another trial where participants used connected devices and biosensors and completed a daily app-based questionnaire:

Those of us who knew it inside and out knew that once you were up and running, it was less than 10 minutes a day to do all of this. But it was overwhelming to the patients. Regardless of how reassuring we were.

Case study interviewee 0053 Vendor

##### Familiarity with study activities and technologies

Some interviewees believed that lack of familiarity with technologies and online interfaces limited recruitment and reduced retention in older target populations. Additionally, interviewees reported that participants disliked wearing devices because they attracted attention. For example, participants in one study disliked wearing a biosensor attached to their chest because of flashing lights that could be seen through their clothing. This transgression of social norms was seen as a disincentive to adherence.

##### Simple interfaces

Many interviewees, including patient representatives, believed that simple DCT interfaces, such as study websites with clear directions, calls to action, and limited text, were essential for retaining participants.

#### Trust

Interviewees held trusting relationships between participants and researchers to be necessary for retention and adherence. They described efforts to maintain this trust through clear communication and frequent contact with remote participants.

##### Communication 

Interviewees felt that DCTs could be particularly vulnerable to misunderstandings by trial participants because there are fewer opportunities to check understanding and explain the rationale behind trial activities:

Yes, I think there certainly are challenges when you don’t have any face-to-face interactions. How do you effectively communicate to participants why it’s important to stay on the routes they were randomised to? We run in a lot with a patient saying, “oh, I am not taking Aspirin anymore, so I need to withdraw from the study.”

Case study interviewee 0016 Trial Staff (management/administration)

##### Maintaining contact

Several interviewees regarded contacting DCT participants (to actively enquire about changes in their condition or circumstances) to be essential for retention:

Following them up every couple of months..has really helped… keeping that continuity with the patients has also been, I think, helpful in the retention of patients. They get sent either a letter or email, or they’re phoned up to ask them, are you still taking your drugs, do you want to change it, has anything medically happened, have you moved?

Case study Interviewee 0012 Trial Staff (management/administration)

Indeed, another interviewee reported that, in a hybrid DCT that ran over several years, people assigned to no treatment or usual treatment had forgotten they were ever enrolled in the trial.

One DCT team that used an interactive study bulletin found this two-way communication extremely popular with participants and felt it encouraged long-term retention and adherence. Some interviewees also maintained contact with participants informally by sending greeting cards or newsletters.

### Involving patients and the public

The ISPOR definition of patient engagement cited earlier clearly envisages patient-researcher interactions as collaboration throughout a research project. However, we observed that interviewees who worked in clinical trials often explained patient and public involvement more narrowly as a tool for achieving participant engagement (defined as successful recruitment, retention, and adherence) rather than as an engagement activity in its own right.

#### Perceived value of DCT participation

Several interviewees said that early patient involvement in their DCT had ensured that the research question was a high priority to potential participants:

Our patient partners came to the kick-off meeting… where they got up on stage, and they told the researchers this is important to us, we really want you guys to work together and figure this out.

Case study interviewee 0016 Trial Staff (management/administration)

One interviewee described how meetings with patients raised unexpected concerns, leading the trial team to modify and refine their protocol to better align with patient priorities:

Before we applied for funding, we had a meeting with the patients’ group. There must have been 20-25 patients, and we talked with them…and you do get some feedback that you don’t expect. It came through that patients were very concerned about amputation and eye disease; they wanted this highlighted as a particular outcome.

Case study interviewee 0041 Trial Staff (clinical/research)

#### Trust

Maximising patient involvement was a key project objective in one DCT, which used socialisation, communication, and education strategies to achieve this. For example, the trial team believed it was important for the patient representatives to feel comfortable together before participating in team meetings. This interviewee described how a research staff member supported this socialisation:

First, she created a very strong environment where it was just the patient partners. She was very protective of that space early on so that they were able to build a rapport amongst themselves but really have the time to orient themselves to research.

Case study interviewee 0016 Trial Staff (management/administration)

##### Communication

Another important role for patient partners in that study was identifying potential barriers to understanding. For example, abbreviations and acronyms were identified as limiting their ability to ask questions and offer opinions. The trial team also aimed to build patient partners’ confidence to participate by providing education on research methods.

## Discussion

Our analysis suggests that people working on DCTs view participant engagement as vital for recruitment, retention, and adherence. Interviewees described various activities encouraging people to enrol and remain in their DCT, many of which were informed by the involvement of patients and the public. In this way, DCTs are no different from contemporary conventional clinical trials, where it is increasingly recognised that patient and public involvement must be addressed throughout the trial process, from promoting general awareness of trial participation opportunities to disseminating results [18]. For this reason, we might expect involvement-informed interventions that improve recruitment, retention, and adherence in conventional trials also to be effective in DCTs. However, DCTs may differ from conventional trials in the degree of emphasis placed on efforts to engender trust between participants and researchers; DCTs are perceived to need greater effort to outweigh the lack of in-person site-based interactions. Additionally, interviewees displayed an awareness that the technologies used to enable DCTs can both increase the burden and increase the value of participation to an individual. This multi-dimensional effect of study design choices makes it likely that adoption of DCT models may have unpredictable effects on recruitment, retention, and adherence.

### Identifying and attracting potential participants

Our interviewees thought that a potential participant’s likelihood of taking part in a DCT was strongly influenced by their beliefs about the value of the research and the trustworthiness of the research team, i.e. if a person believed the trial was about something important, personally relevant and run by a trustworthy organisation, they would be more predisposed to participate. Therefore, targeting people more likely to fit these criteria was seen as worthwhile. In DCTs without site-based recruitment, this can require the use of routinely collected healthcare data or social media targeted advertising. In addition, early involvement of patient groups was regarded as a mechanism for identifying the likely priorities of potential participants.

A further consideration for potential participants is the perceived burden of DCT participation. Using familiar technologies and offering a choice of participation modes may broaden cohort diversity and improve recruitment.

### Retaining participants and encouraging their adherence to trial activities

Our analysis suggests that, while distinct from recruitment, the factors influencing retention and adherence in DCTs are related to the same underlying motivators: personal values and circumstances. Interviewees believed that problems with adherence and retention in their trials were due to aspects of trial conduct that conflicted with a participant’s initial positive attitude towards the research. In other words, the experience of participation did not align with what motivated an individual to take part. Thus, maintaining adherence and retention is, to an extent, dependent on how the research purpose and critical trial information were communicated during recruitment. Again, input from patients and the public can assist in developing recruitment materials and ensuring that within-study communications align with them.

Several interviewees felt that two-way communication between participants and trial personnel was necessary to ensure participants understood trial requirements and to build trusting relationships.

The ability of technology-enabled DCTs to give participants helpful or interesting information they would not otherwise be able to access can increase the perceived value of participation. Near real-time data monitoring was also seen as a way for DCT teams to check that participants were engaging as intended. However, if this process triggers only automated reminders, it may diminish a sense of connectedness that an individual participant feels is essential to their participation.

Simplifying required participant activities should reduce the burden of trial participation [19], for example, minimising the number of devices and technological interfaces involved in data collection and uploading. However, oversimplifying the participation process may have the unintended consequence of reducing the perceived relevance of trial activities. It should be clear to participants why actions are required and what behind-the-scenes activity, such as linkage to routine healthcare data, is being done to achieve the study’s aims. User testing, with patient or public involvement groups, or pilot testing of technological platforms and processes, may help identify opportunities to simplify processes or areas where additional attention may be needed to explain how the trial works. Trial teams must also ensure that wearable devices do not engender a social burden by inducing self-consciousness.

Even when the experience of trial participation does not conflict with the original motivators, changes in a participant’s circumstances or values, increasingly likely during long-duration trials, may result in them choosing not to adhere to study activities or withdrawing entirely. DCTs may limit such withdrawals or missing data by offering a degree of flexibility in participation. For example, participants may choose to continue to allow researchers access to routinely collected healthcare data for primary outcome ascertainment but temporarily opt-out of completing online questionnaires.

### Involving patients and the public

**Fig. 1:**
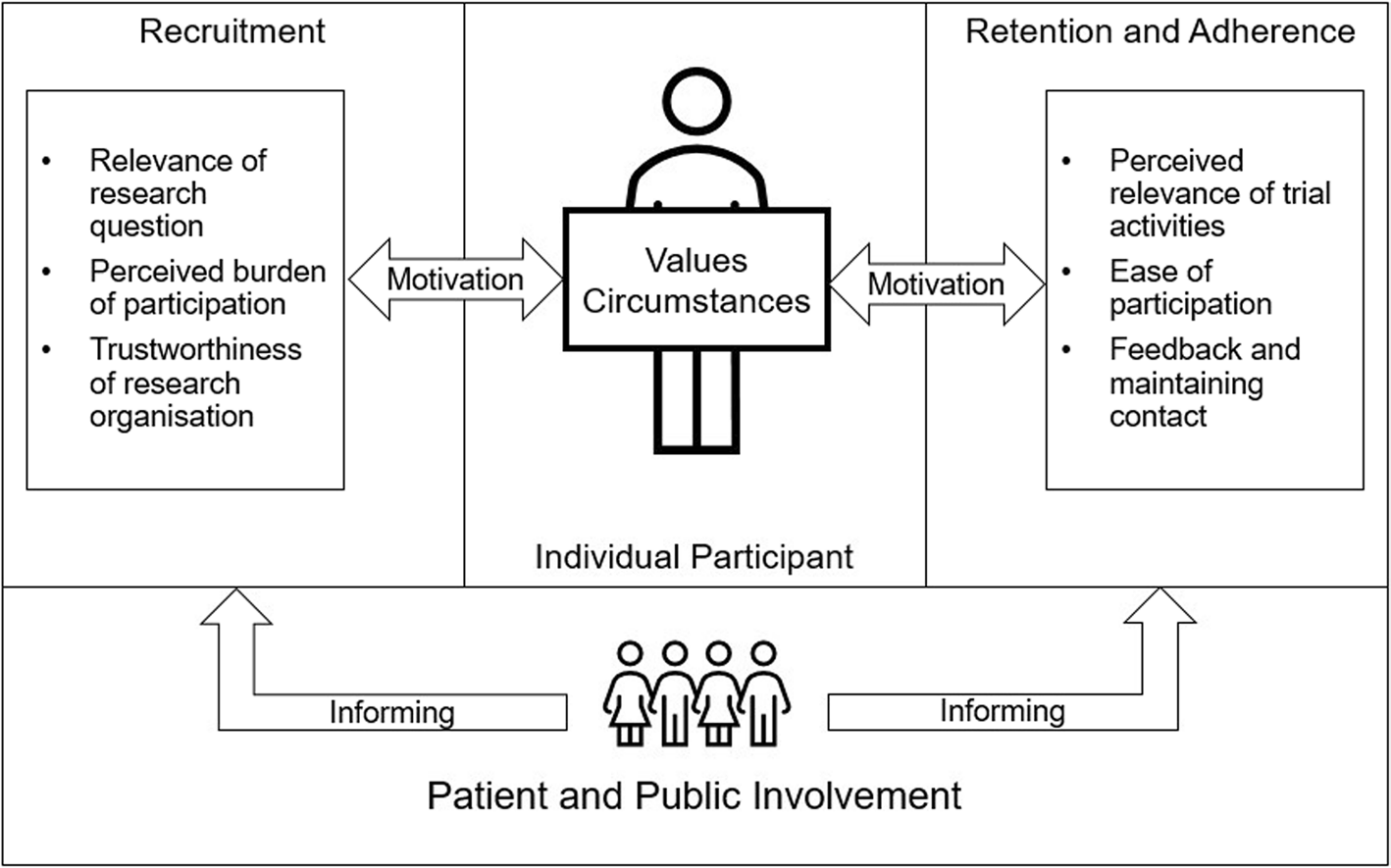
Schematic representation of a participant-centred view of DCT recruitment, retention, and adherence

Our thematic analysis of stakeholder views suggests that patient and public input is believed by trial staff to inform successful participant engagement in DCTs by facilitating alignment of trial communications and activities with participants’ values and circumstances. We have illustrated this conceptual relationship in Fig. 1.

### Limitations

The limitations of the methodology used here to explore stakeholder views of DCTs have been previously described [1]. A significant additional challenge to this analysis was variations in what interviewees meant and understood by engagement. For example, interviewees frequently used patient engagement, or involvement, interchangeably with participant engagement, meaning recruitment, retention, and adherence. In general, stakeholders in the public sector tended to use the term to describe either public engagement activities (such as awareness campaigns) or activities aimed at improving retention or adherence. In contrast, commercial stakeholders often used the word to describe the processes of identifying, recruiting, and enrolling potential participants. We, therefore, focused on specific activities rather than terminology. Where an interviewee used the word “engagement”, we tried to define what they meant; as this was a secondary analysis, we could not confirm these assumptions. A further limitation of our data was that we could not interview as many DCT-experienced patient representatives as initially planned, thus limiting generalisability.

## Conclusions

As in conventional site-based trials, DCT recruitment, retention, and adherence are affected by many factors, some of which may be amenable to behaviour-theory informed interventions. Our analysis emphasises the role of patient and public involvement in optimising efforts to promote participant engagement in DCTs. Consultation with patients and the public can provide insight into what is likely to be important to potential participants and whether planned DCTs are suitable. Such input can help shape the research question and trial design to appeal to potential participants and ensure trial materials and websites are pitched correctly and include necessary information. Without site-based personal interaction between researchers and participants, the importance of these study communications is enhanced. Dialogue with current study participants may improve trust and help people remain in a trial; it can also provide insights into the participation experience and ways to improve it. DCT developers should therefore seek ways to facilitate dialogue between participants and researchers. Finally, further research will be needed to support optimal recruitment, retention, and adherence strategies for DCTs. Qualitative research embedded in DCTs could explore participants’ experiences and perceptions, guiding future research design.

## Data Availability

The dataset is the in-depth interview transcripts. Access is restricted to the study team in accordance with the requirements of the ethical approval.

## Abbreviations

DCT: Decentralised clinical trial
COREQ: Consolidated criteria for reporting qualitative research
EFPIA: European Federation of Pharmaceutical Industries and Associations
EU: European Union
IMI: Innovative Medicines Initiative
ISPOR: International Society for Pharmacoeconomics and Outcomes Research
NHS: National Health Service
